# Missplicing due to a synonymous, T96= exonic substitution in the T-box transcription factor *TBX19* resulting in isolated ACTH deficiency

**DOI:** 10.1530/EDM-21-0128

**Published:** 2021-09-03

**Authors:** Ashwini Maudhoo, Avinaash Maharaj, Federica Buonocore, Gabriel Angel Martos-Moreno, Jesús Argente, John C Achermann, Li F Chan, Lou A Metherell

**Affiliations:** 1 Centre for Endocrinology, William Harvey Research Institute, Barts and the London School of Medicine and Dentistry, Queen Mary University of London, London, UK; 2 Genetics and Genomic Medicine, UCL Great Ormond Street Institute of Child Health, London, UK; 3 Department of Endocrinology, Hospital Infantil Universitario Niño Jesús, Instituto de Investigación La Princesa, Madrid, Spain; 4 Department of Pediatrics, Universidad Autónoma de Madrid, Madrid, Spain; 5 Centro de Investigación Biomédica en Red de Fisiopatología de la Obesidad y Nutrición (CIBEROBN), Instituto de Salud Carlos III, Madrid, Spain; 6 CEI UAM + CSIC, IMDEA Food Institute, Madrid, Spain

**Keywords:** Neonatal, Male, White, United Kingdom, Pituitary, Adrenal, Paediatric endocrinology, Genetics and Mutation, Insight into disease pathogenesis or mechanism of therapy, September, 2021

## Abstract

**Summary:**

Congenital isolated ACTH deficiency (IAD) is a rare condition characterised by low plasma ACTH and serum cortisol with normal production of other pituitary hormones. TBX19 (also known as TPIT) is a T-box pituitary restricted transcription factor important for *POMC* gene transcription and terminal differentiation of POMC-expressing cells. *TBX19* gene mutations have been shown to cause neonatal-onset congenital IAD. We report a neonate of Romanian origin, who presented at 15 h of life with respiratory arrest and hypoglycaemia which recurred over the following 2 weeks. Biochemical investigations revealed IAD, with undetectable serum cortisol (cortisol < 1 μg/dL; normal range (NR): 7.8–26.2) and plasma ACTH levels within the normal range (22.1 pg/mL; NR: 4.7–48.8). He responded to hydrocortisone treatment. Patient DNA was analysed by a HaloPlex next-generation sequencing array targeting genes for adrenal insufficiency. A novel homozygous synonymous mutation p.Thr96= (Chr1:168260482; c.288G>A; rs376493164; allele frequency 1 × 10^−5^, no homozygous) was found in exon 2 of the *TBX19* gene. The effect of this was assessed by an *in vitro* splicing assay, which revealed aberrant splicing of exon 2 giving rise to a mutant mRNA transcript whereas the WT vector spliced exon 2 normally. This was identified as the likely cause of IAD in the patient. The predicted protein product would be non-functional in keeping with the complete loss of cortisol production and early presentation in the patient.

**Learning points:**

- Synonymous variants (a nucleotide change that does not alter protein sequence) usually thought to be benign may still have detrimental effects on RNA and protein function causing disease. Hence, they should not be ignored, especially if very rare in public databases.
- *In vitro* splicing assays can be employed to characterise the consequence of intronic and exonic nucleotide gene changes that may alter splicing.
- Establishing a diagnosis due to a *TBX19* mutation is important as it defines a condition of isolated ACTH deficiency not associated with additional pituitary deficiencies.

## Background

Congenital isolated ACTH deficiency (IAD) is a rare cause of secondary adrenal insufficiency characterised by low plasma ACTH and serum cortisol in the setting of otherwise preserved anterior pituitary function. Neonatal onset of the disease can be caused by mutations in the T-box transcription factor 19 (*TBX19* or *TPIT*) while no genetic aetiology has yet been determined for later onset (1, 2, 3).

*TBX19* is a T-box pituitary restricted transcription factor involved in *POMC* gene transcription and terminal differentiation of POMC-expressing cells. To date, 29 mutations in *TBX19* have been annotated by the Human Gene Mutation Database (http://www.hgmd.cf.ac.uk) in association with IAD, including many missense changes, large and small deletions and four mutations, which occur within canonical splice site motifs and are predicted to affect splicing.

Here, we report a congenital IAD case with a novel, synonymous, exonic *TBX19* mutation, NM_005149.3:c.288G>A (p.T96=), resulting in missplicing.

## Case presentation

A neonate of Romanian origin presented at 15 h of life with respiratory arrest and hypoglycaemia. He was born of non-consanguineous parents by vaginal delivery following an uneventful pregnancy. He suffered from jaundice on the third day of life for which he had 72 h of phototherapy. Over the following 2 weeks, recurrent hypoglycaemia was documented. On examination, he had normal male genitalia and no abnormal pigmentation. Biochemical investigations, whilst hypoglycaemic, revealed the absence of hyperinsulinism, normal free T4 and adequate GH levels. Serum cortisol was undetectable (cortisol < 1 μg/dL; NR: 7.8–26.2) and plasma ACTH concentrations were within the normal range (22.1 pg/mL; NR: 4.7–48.8) in the presence of documented hypoglycaemia (Table 1). The ACTH concentrations are inappropriate and abnormal in the setting of undetectable serum cortisol. At 1 month of age, he was diagnosed with suspected IAD. He responded to hydrocortisone treatment and continues on replacement. He had normal growth (height 90th percentile) without obesity (BMI 25th percentile) at 3.5 years of age. Standard short Synacthen (ACTH_1–24_) testing at this age showed a completely absent cortisol response to ACTH stimulation (<1 μg/dL) during euglycaemia. Repeat plasma ACTH was low (3.85 pg/mL) at this point. He has a healthy sister who is 30 months older. There is a family history of adrenal disease as his maternal aunt has been on hydrocortisone treatment from 18 months of age with a diagnosis of suspected IAD.

**Table 1:** Biochemical results.

Analysis	18 days of life	Age 3.5 years	Reference range
Glucose, mg/dL	35	76	42–100
Insulin, μIU/mL	3.3	1.6	4–11
Urea, mg/dL	1.00	22	8.00–35.00
Creatinine, mg/dL	0.3	0.4	0.32–1.06
Total bilirubin, mg/dL	10.4	0.5	0.2–0.8
AST, U/L	32	34	27–80
ALT, U/L	15	16	20–66
ALP, U/L	182	116	37–221
Sodium, mEq/L	136	138	133–141
Potassium, mEq/L	5.6	4.6	4.3–6.2
Lactic acid, mg/dL	33.54	–	6.5–20
GH, ng/mL	14.4	–	>7
IGF-I, ng/mL	–	87	33–209
IGFBP-3, μg/mL	–	2.16	1.17–4.17
17-OHP, ng/mL	–	–	0.1–1.48
ACTH, pg/mL	19	3.85	4.7–48.8
Cortisol, μg/dL	<1	<1	7.8–26.2
Peak cortisol post ACTH stimulation	–	<1	

## Investigation

### Materials and methods

#### DNA extraction

DNA was extracted from samples of the patient’s blood leukocytes using the GE Healthcare Illustra Nucleon Genomic DNA Extraction Kit.

#### Next generation sequencing HaloPlex

The patient’s DNA was analysed using a custom HaloPlex DNA target enrichment panel (Agilent Technologies Inc), designed to capture 160 known and candidate genes involved in adrenal development and function, thereby identifying potential variants in genes of interest (4). Captured genes were subsequently sent for sequencing on a MiSeq next-gen sequencer. Sequence alignment and variant calling were performed using SureCall software (version 2.0; Agilent Technologies Inc) and the resulting variant call files (vcf) were uploaded to both SureCall and Ingenuity Variant Analysis (IVA) (QIAGEN Bioinformatics; www.ingenuity.com) (4). Variants were filtered assuming an autosomal recessive mode of inheritance through the pipeline in Supplementary Fig. 1 (see section on supplementary materials given at the end of this article) (4). Confirmation of the *TBX19* variant was performed by Sanger sequencing using specifically designed primers; 5’-TTGAGAGCTTGTTTGGAGG-3’ and 5’-GCGCTCGGTCGAAGA-3’.

#### *In silico* predictions

A number of *in silico* tools were used to predict the mRNA structure and the pathogenicity of the variant. Minor allele frequency (MAF) was determined from the Genome Aggregation Database (gnomAD) browser and the variant effect was predicted with common algorithms such as SIFT (Sorting Intolerant from Tolerant: http://www.blocks.fhcrc.org/sift/SIFT.html), PROVEAN (Protein Variation Effect Analyzer: http://provean.jcvi.org/index.php), SNAP2 (https://www.rostlab.org/services/snap/) and MutationTaster (http://www.mutationtaster.org/). Splice function was assessed using the Human Splicing Finder (HSF: http://www.umd.be/HSF3/) software.

#### *In vitro* splicing assay

An *in vitro* splicing assay was designed using the commercially obtained pET01 Exon-trap vector (MoBiTec GmbH, Germany) to allow selective cloning of a DNA fragment of interest. WT and mutant exon 2 of *TBX19* and flanking intronic sequence were amplified using a standard PCR protocol and specifically designed primers, 5’-AGCTATATCTAGATTGGGGTTGTGGACT-3’ and 5’-AGCTATATCTAGAAATGCACCCTTGATATT-3’, containing the restriction enzyme sequence for *Xba*I. The amplified products were gel purified and cloned into the polylinker with subsequent transfection of mutant and WT vectors into HEK293T cells. If the insert expressed an exon in the correct orientation, RNA was generated from the vector with removal of intronic sequences. RT of mRNA was then achieved with subsequent DNA amplification as previously described using primers 5’-GCGAAGTGGAGGATCCACAAG-3’ and 5’-ACCCGGATCCAGTTGTGCCA-3’ targeting the pET01 vector (5). Resulting amplification products were run on a gel and purified for subsequent Sanger sequencing.

### Results

**Figure 1:**
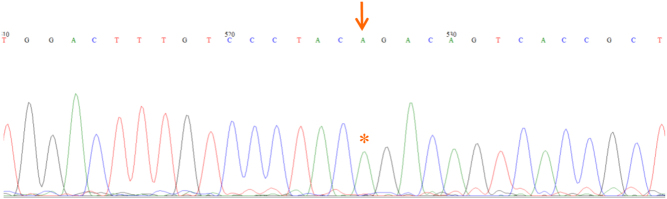
Partial chromatogram showing the homozygous variant present in the patient. G>A change at position c.288 is indicated with a red arrow and asterisk.

A synonymous, homozygous variant (g.1–168260482G>A; rs376493164; c.288G>A (ENST00000367821.3); p.T96=) was identified in *TBX19* and confirmed by Sanger sequencing (Fig. 1). No other variants of relevance were found in *TBX19* or other genes. Both parents were heterozygous for the change, fitting an autosomal recessive mode of inheritance of the variant. The variant was very rare, with a MAF of 1.06e−5 in gnomAD, and no homozygotes reported. The resulting change was predicted to be benign by SIFT (0.37, tolerated), PROVEAN (0.00, neutral protein) and SNAP2 (–61, neutral protein) but ‘disease causing with splice site changes' by MutationTaster. The latter was supported by analysis using the HSF 3.0 software, which predicted a new splice acceptor site with a consensus value (CV) of 92.39, with strong sites having a CV of > 80. This is predicted to result in the loss of 86bp from the mRNA, giving rise to a frameshift, early stop codon and thus a truncated protein (p.R69Qfs*67). These alleles are predicted to be liable to nonsense-mediated mRNA decay (NMD).

**Figure 2:**
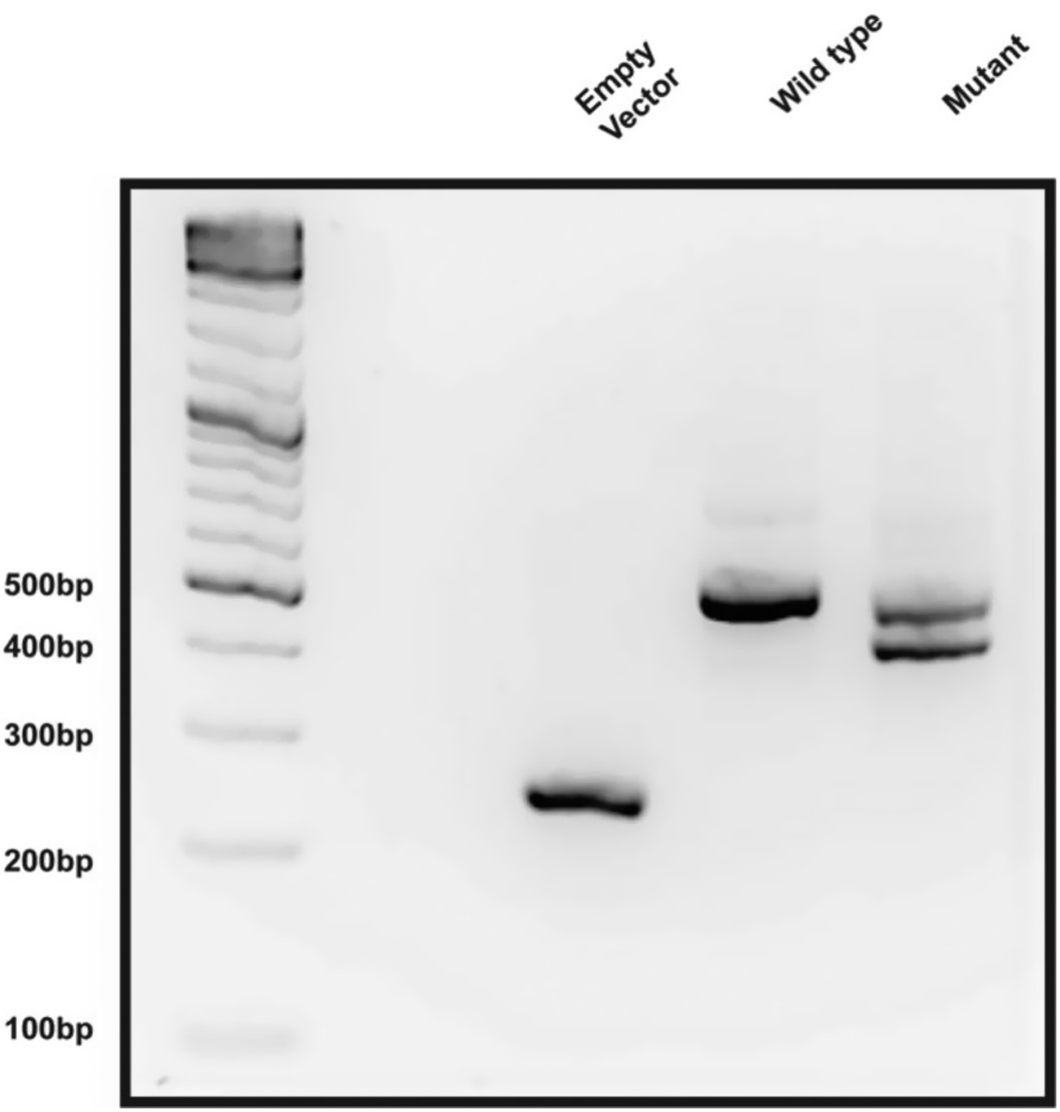
Representative image of gel electrophoresis of PCR products from *TBX19* splicing assay (*n* = 3).

The effect of the mutation was assessed by an *in vitro* splicing assay, using the pET01 ExonTrap cloning vector (MobiTec) transfected into HEK293 cells, comparing WT and mutant heterologous minigenes. The WT vector spliced exon 2 normally, giving a single band of the expected size (500 bp) whereas the mutant minigene had aberrant splicing giving rise to two bands one of approximately 500 bp and one smaller, consistent with a product 86 bp shorter (Fig. 2).

**Figure 3:**
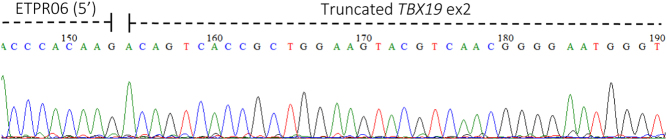
Partial chromatogram of sequence from the smaller band in the mutant sample showing aberrant splicing in the middle of exon 2.

**Figure 4:**
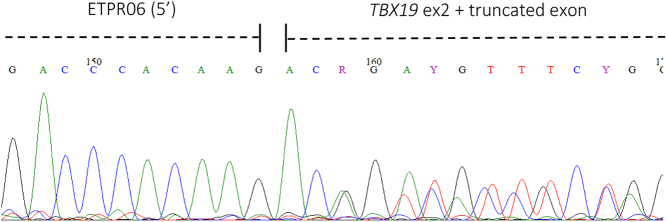
Partial chromatogram of sequence from the larger band in the mutant sample shows sequences for both the normally spliced exon and splicing in the middle of exon 2 suggesting a hybrid of the two sequences.

**Figure 5:**
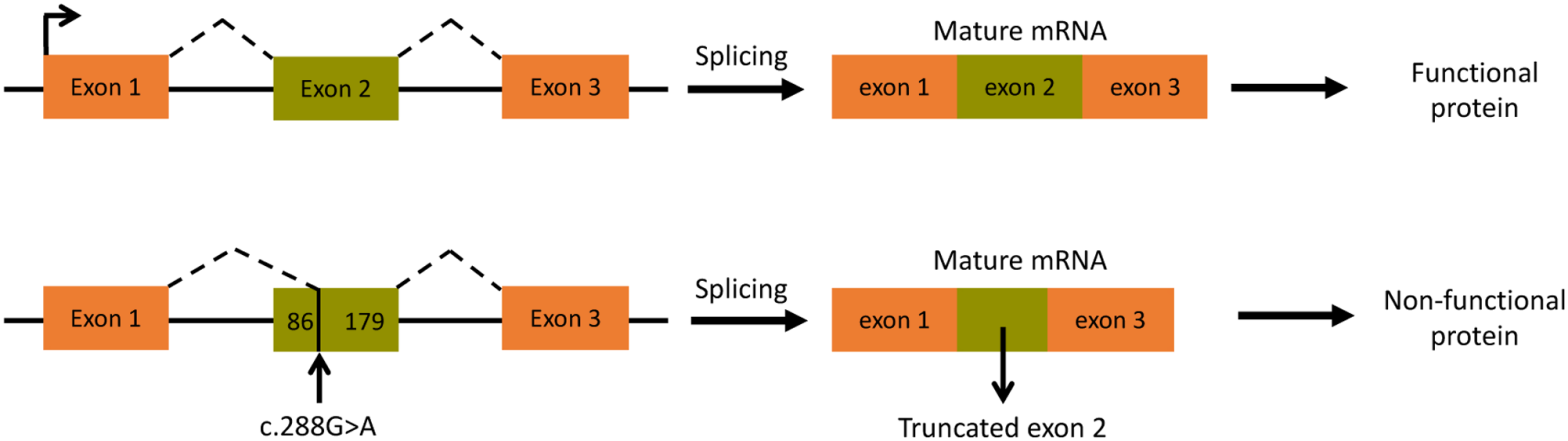
Representation of the effect of the *TBX19* splice site mutation, the upper panel represents normal splicing while the lower panel indicates the outcome from the c.288G>A variant in exon 2.

The band from the WT and the two bands from the mutant sample (Fig. 2) were excised from the gel, purified and sent for Sanger sequencing (Figs 3, 4 and 5). This confirmed that the aberrant splice site resulted in the production of a truncated transcript missing 86 bp but normal splicing also occurs resulting in the full-length transcript (Figs 3, 4 and 5), in approximately 1:1 ratio. The WT spliced exon 2 normally.

The c.288G>A variant in exon 2 creates a new splice acceptor site which permits splicing 86 bp into exon 2 resulting in a frameshift mutation and early termination of the protein if the transcript is not eliminated by nonsense-mediated mRNA decay (Fig. 5).

## Outcome and follow-up

In this case, the clinical picture of complete loss of cortisol production and early onset disease suggests *TBX19* as the causative gene and the finding that the synonymous T96= *TBX19* change causes aberrant splicing makes it the likely pathogenic mutation in this individual.

## Discussion

Common presentations of congenital IAD due to *TBX19* mutations have been reported to include hypoglycaemia, seizures and jaundice (2). The initial descriptions of the disease reported homogeneous phenotypic features for patients with *TBX19* mutations. However, as we learn more about the disease, different genotype–phenotype relationships have been observed with presentations such as recurrent respiratory infections as well as Chiari type 1 malformation, tall stature and dysmorphic features (6, 7). Differences in presentations emphasise the need for genetic characterisation as unrecognised and untreated congenital IAD can be life-threatening.

Sanger sequencing, which set the foundation for sequence-driven research, has been supplanted by next-generation sequencing (NGS) technologies, allowing the concomitant detection of mutations in many genes. The use of actual or virtual panels can allow a more focused sequencing analysis of disease-related regions. In our study, using a targeted NGS HaloPlex panel, we detected a synonymous variant in *TBX19* suspected to be causative since it was homozygous and extremely rare in population databases such as gnomAD.

HaloPlex is an amplicon-based method in which specific regions of the genome are amplified through PCR, thus achieving better coverage of guanine/cytosine-rich regions and higher read depths. The ability to better focus on a controlled number of disease-related regions allows for more focused sequencing and improved coverage.

Previously described splice mutations in *TBX19* are all within canonical splice site motifs, and while untested, are predicted to cause complete skipping of the relevant exon. Comparatively, the variant found in this patient leads to the production of a normally spliced transcript and one lacking 86 bp of exon 2 and might thus be expected to cause a milder phenotype. However, the patient shows a classical presentation suggesting that *in vivo*, the mutant truncated transcript may predominate.

Interestingly, plasma ACTH levels in our patient were within normal range indicative of some secretion capability from pituitary corticotrophs. However, the ACTH concentrations are completely inappropriate in the context of overt hypocortisolaemia and hypoglycaemia. This is consistent with previous reports of patients with *TBX19* variants whereby plasma ACTH can vary from undetectable to 'normal' ranges (2). In such cases, we would anticipate that at basal levels (in the absence of hypocortisolaemia) plasma ACTH concentrations are insufficient to maintain adrenal gland growth and zona fasciculata differentiation resulting in IAD. In the *Tpit* knock-out mouse model, inactivation of the *Tpit* gene results in loss of POMC expression in corticotropes and adrenal hypoplasia (1). Interestingly, a recent report shows a reduction in pituitary cell size and organelle content in *Tpit^−/−^* animals rather than cell number (8).

The *in vitro* splicing assay of the mutant sample showed the production of a normal transcript and a truncated version, with an apparent 1:1 ratio of large to small band (Fig. 2). The normal transcript is seen because although the variant creates a new cryptic splice acceptor site with a high score, it does not alter the canonical acceptor site for exon 2, hence both splicing events are taking place in the patient, reducing the quantity of normal transcript to a level that presumably does not support sufficient/effective corticotrope function. The smaller band consisted purely of truncated transcript whereas the upper band appears to be a mixture of the two in approximately equal measure (Figs 3 and 4, respectively). Therefore, we might predict that at least 75% of the transcript produced will be truncated and consequently non-functional. If the transcript is not destroyed by nonsense-mediated mRNA decay, a process commonly occurring when a premature termination codon is present, the resulting protein will be p.R69Qfs*67. TPIT has a T-box domain spanning amino acids 40–223, most of which will be absent/altered in the truncated protein produced. There is also a known disease mutation at this position (HGMD CM043105) implicated in early onset IAD (3).

Only one of the four computational predictors interpreted this variant as disease causing. This highlights the current shortcomings of *in silico* prediction tools and the need for functional evaluation of predicted rare synonymous changes at both mRNA and protein level (5, 6).

While *in silico* tools for predicting the functional effects of nonsynonymous variants are well-substantiated, when it comes to synonymous variants, the results are often inconclusive. Despite synonymous single nucleotide variants (sSNV) being as prevalent as non-synonymous SNVs and also equally pathogenic as supported by the literature, there is a paucity of experimental studies investigating their functional effects (9). This may be the limiting factor in the development of reliable prediction tools for synonymous variants. Synonymous variants have been implicated in a variety of diseases through alteration of transcription, mRNA stability and splicing (10). Importantly, they may modulate gene expression by disrupting transcription and pre-mRNA splicing or regulators within protein-coding regions (9). This makes the work-up of synonymous variants vital, especially when found in known causative genes and when very rare in public databases.

## Supplementary Material

Supplementary Figure 1. Filtration strategy for variant screening from HaloPlex data.

Supplementary Figure 2 A. Sequence expected if normal splicing occurs in TBX19 exon 2. B. p.T96= variant in TBX19 exon 2 (depicted in red) creates a new alternative splice acceptor site. C. Expected sequence if the cryptic splice site is used in TBX19 exon 2.

## Declaration of interest

The authors declare that there is no conflict of interest that could be perceived as prejudicing the impartiality of the research reported.

## Funding

This work was supported by the Medical Research Council (grant number MR/K020455/1), Barts Charity (grant number MGU0438). L F C received funding from International Fund for research on Congenital Adrenal Hyperplasia (IFCAH), British Society of Paediatric Endocrinology and Diabetes (BSPED), Barts and the London Charity (MGU0458) and Medical Research Council (MRC) UK/Academy of Medical Sciences Fellowship Grant G0802796. J C A received funding from The Wellcome Trust (209328/Z/17/Z).

## Patient consent

Written informed consent for publication of this case report was obtained from the patient’s parents.

## Author contribution statement

A M, A M and F B conducted the experiments and analyses. G A M and J A phenotyped the patient. J C A, L F C and L A M conceived the study and supervised the work. All authors contributed to the writing of the manuscript.
