# Supplementary figures and images for: Missplicing due to a synonymous, T96= exonic substitution in the T-box transcription factor TBX19 resulting in isolated ACTH deficiency
Source: Endocrinol Diabetes Metab Case Rep. 2021 Sep 3;2021:21-0128. doi: 10.1530/EDM-21-0128 (PMC8495723; doi:10.1530/EDM-21-0128)

### Supplementary Figure 1.

Filtration strategy for variant screening from HaloPlex data.

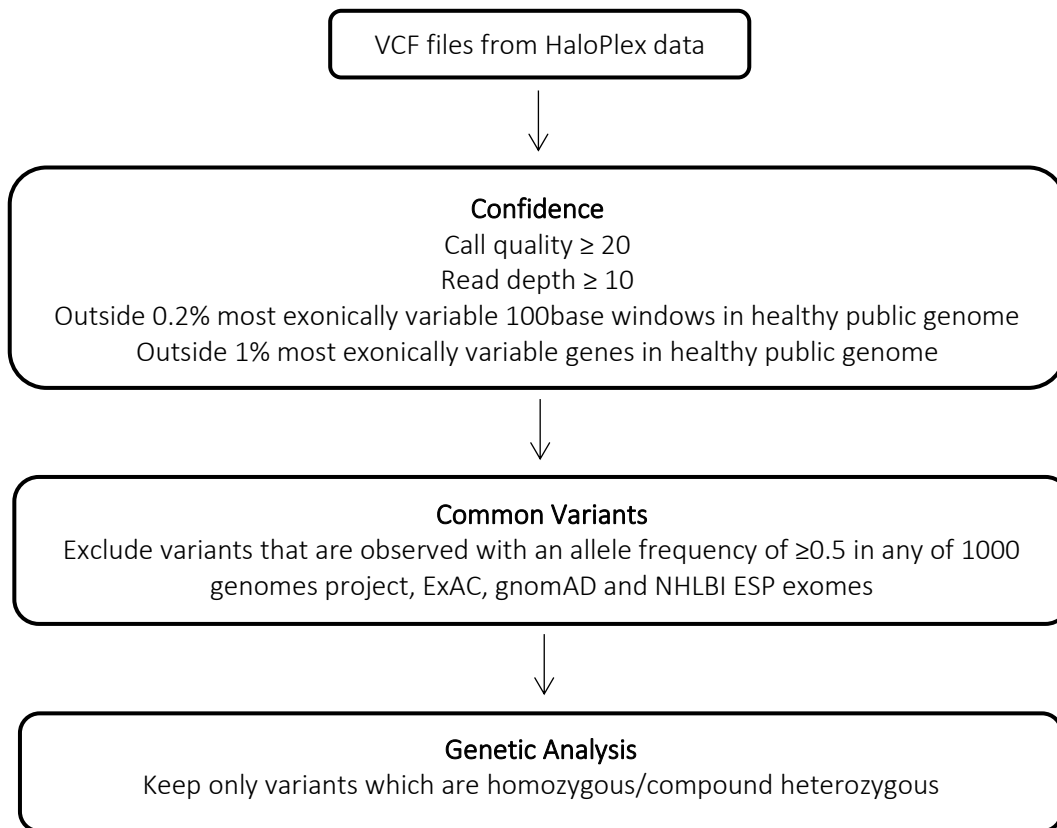

Supplement: Supplementary Figure 1. Filtration strategy for variant screening from HaloPlex data. [file supplementary_figure_1.pdf]
