# Supplementary material for: Missplicing due to a synonymous, T96= exonic substitution in the T-box transcription factor TBX19 resulting in isolated ACTH deficiency
Source: Endocrinol Diabetes Metab Case Rep. 2021 Sep 3;2021:21-0128. doi: 10.1530/EDM-21-0128 (PMC8495723; doi:10.1530/EDM-21-0128)

Supplementary Figure 2

A. Sequence expected if normal splicing occurs in *TBX19* exon 2.

B. p.T96= variant in *TBX19* exon 2 (depicted in red) creates a new alternative splice acceptor site.

C. Expected sequence if the cryptic splice site is used in *TBX19* exon 2.

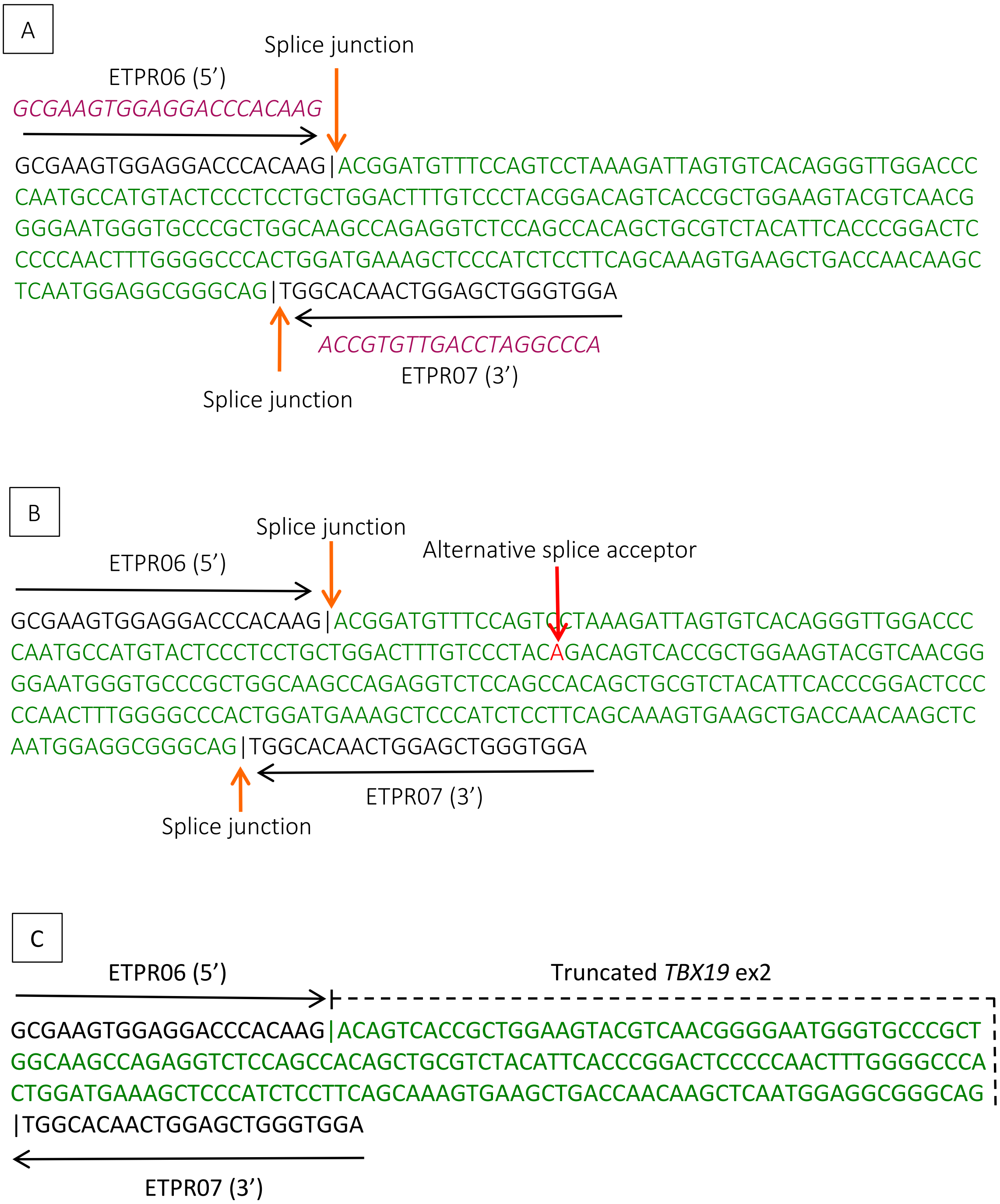

Supplement: Supplementary Figure 2 A. Sequence expected if normal splicing occurs in TBX19 exon 2. B. p.T96= variant in TBX19 exon 2 (depicted in red) creates a new alternative splice acceptor site. C. Expected sequence if the cryptic splice site is used in TBX19 exon 2. [file supplementary_figure_2.pdf]
